# Atrophy in the Thalamus But Not Cerebellum Is Specific for *C9orf72* FTD and ALS Patients – An Atlas-Based Volumetric MRI Study

**DOI:** 10.3389/fnagi.2018.00045

**Published:** 2018-03-15

**Authors:** Sonja Schönecker, Christiane Neuhofer, Markus Otto, Albert Ludolph, Jan Kassubek, Bernhard Landwehrmeyer, Sarah Anderl-Straub, Elisa Semler, Janine Diehl-Schmid, Catharina Prix, Christian Vollmar, Juan Fortea, Deutsches FTLD-Konsortium, Carola Roßmeier, Hans-Jürgen Huppertz, Thomas Arzberger, Dieter Edbauer, Berend Feddersen, Marianne Dieterich, Matthias L. Schroeter, Alexander E. Volk, Klaus Fließbach, Anja Schneider, Johannes Kornhuber, Manuel Maler, Johannes Prudlo, Holger Jahn, Tobias Boeckh-Behrens, Adrian Danek, Thomas Klopstock, Johannes Levin

**Affiliations:** Department of Neurology, University of Ulm, Ulm, Germany; ^1^Department of Neurology, Ludwig Maximilians Universität München, Munich, Germany; ^2^Department of Neurology, University of Ulm, Ulm, Germany; ^3^Department of Psychiatry and Psychotherapy, Technical University of Munich, Munich, Germany; ^4^Hospital San Pau Barcelona, Barcelona, Spain; ^5^Swiss Epilepsy Clinic, Klinik Lengg, Zurich, Switzerland; ^6^Center for Neuropathology and Prion Research, Ludwig Maximilians Universität München, Munich, Germany; ^7^German Center for Neurodegenerative Diseases (DZNE), Munich, Germany; ^8^Institute for Metabolic Biochemistry, Ludwig Maximilians Universität München, Munich, Germany; ^9^Munich Cluster for Systems Neurology (SyNergy), Munich, Germany; ^10^Department of Palliative Medicine, Ludwig Maximilians Universität München, Munich, Germany; ^11^Max Planck Institute for Human Cognitive and Brain Sciences (MPG), Leipzig, Germany; ^12^Clinic for Cognitive Neurology, University Hospital Leipzig, Leipzig, Germany; ^13^Institute of Human Genetics, University Medical Center Hamburg-Eppendorf, Hamburg, Germany; ^14^German Center for Neurodegenerative Diseases (DZNE), Bonn, Germany; ^15^Department for Neurodegenerative Diseases and Geriatric Psychiatry, University Hospital Bonn, Bonn, Germany; ^16^Department of Psychiatry and Psychotherapy, Friedrich-Alexander University Erlangen-Nürnberg, Erlangen, Germany; ^17^Department of Neurology, Rostock University Medical Center, Rostock, Germany; ^18^German Center for Neurodegenerative Diseases (DZNE), Rostock, Germany; ^19^Department of Psychiatry and Psychotherapy, University Medical Center Hamburg-Eppendorf, Hamburg, Germany; ^20^AMEOS Klinikum Heiligenhafen, Heiligenhafen, Germany; ^21^Department of Diagnostic and Interventional Neuroradiology, Technical University of Munich, Munich, Germany; ^22^Friedrich Baur Institute at the Department of Neurology, Ludwig Maximilians Universität München, Munich, Germany

**Keywords:** C9orf72, frontotemporal dementia, amyotrophic lateral sclerosis, atlas based volumetric MRI analysis, thalamus, cerebellum, salience network

## Abstract

**Background:** The neuropathology of patients with frontotemporal dementia (FTD) or amyotrophic lateral sclerosis (ALS) due to a *C9orf72* mutation is characterized by two distinct types of characteristic protein depositions containing either TDP-43 or so-called dipeptide repeat proteins that extend beyond frontal and temporal regions. Thalamus and cerebellum seem to be preferentially affected by the dipeptide repeat pathology unique to *C9orf72* mutation carriers.

**Objective:** This study aimed to determine if mutation carriers showed an enhanced degree of thalamic and cerebellar atrophy compared to sporadic patients or healthy controls.

**Methods:** Atlas-based volumetry was performed in 13 affected *C9orf72* FTD, ALS and FTD/ALS patients, 45 sporadic FTD and FTD/ALS patients and 19 healthy controls. Volumes and laterality indices showing significant differences between mutation carriers and sporadic patients were subjected to binary logistic regression to determine the best predictor of mutation carrier status.

**Results:** Compared to sporadic patients, mutation carriers showed a significant volume reduction of the thalamus, which was most striking in the occipital, temporal and prefrontal subregion of the thalamus. Disease severity measured by mini mental status examination (MMSE) and FTD modified Clinical Dementia Rating Scale Sum of Boxes (FTD-CDR-SOB) significantly correlated with volume reduction in the aforementioned thalamic subregions. No significant atrophy of cerebellar regions could be detected. A logistic regression model using the volume of the prefrontal and the laterality index of the occipital subregion of the thalamus as predictor variables resulted in an area under the curve (AUC) of 0.88 while a model using overall thalamic volume still resulted in an AUC of 0.82.

**Conclusion:** Our data show that thalamic atrophy in *C9orf72* mutation carriers goes beyond the expected atrophy in the prefrontal and temporal subregion and is in good agreement with the cortical atrophy pattern described in *C9orf72* mutation carriers, indicating a retrograde degeneration of functionally connected regions. Clinical relevance of the detected thalamic atrophy is illustrated by a correlation with disease severity. Furthermore, the findings suggest MRI volumetry of the thalamus to be of high predictive value in differentiating *C9orf72* mutation carriers from patients with sporadic FTD.

## Introduction

Frontotemporal dementia (FTD) and amyotrophic lateral sclerosis (ALS) are heterogeneous neurodegenerative disorders that are associated with one another in approximately 15% of the cases (Lomen-Hoerth et al., 2002). FTD can present with socially inappropriate behavior, apathy, lack of empathy, changes in diet and compulsive behaviors. Compared to Alzheimer’s disease there is typically a relative preservation of memory and visuospatial function (Perry and Miller, 2013). ALS is a motor neuron disease that is characterized by progressive degeneration of upper and lower motor neurons. It typically manifests with progressive muscle weakness, muscular atrophy, spasticity and fasciculations (Lomen-Hoerth et al., 2002). The most common known cause of familial FTD, familial ALS or patients with a mixed presentation of both diseases (FTD/ALS) is a hexanucleotide expansion mutation in a non-coding region of *C9orf72* (Cruts et al., 2013). Compared to sporadic patients *c9orf72* mutation carriers have a greater frequency of psychotic symptoms like delusions, hallucinations or paranoid ideation and show more severe memory impairment (Snowden et al., 2015).

Previous neuroimaging studies on *c9orf72* mutation carriers have shown relatively symmetrical atrophy most prominent in the frontotemporal cortex and the insula, in keeping with the atrophy pattern described in sporadic patients (Boxer et al., 2011; Mahoney et al., 2012; Whitwell et al., 2012). In contrast to sporadic patients, however, *c9orf72* mutation carriers appear to have more parietal and occipital cortical atrophy creating a more diffuse cortical atrophy pattern. Furthermore bithalamic and cerebellar involvement have been described (Yokoyama and Rosen, 2012; Bede et al., 2013; Prado et al., 2015; Floeter et al., 2016). Moreover, volumetric imaging data from the genetic frontotemporal dementia initiative (GenFI) show an early affection of thalamus and cerebellum in *C9orf72* mutation carriers compared to healthy controls (HC) as well as to *GRN* and *MAPT* mutation carriers (Rohrer et al., 2015).

A neuropathological hallmark of *C9orf72* mutation carrier status is the intracellular deposition of five dipeptide repeat proteins (DPR). These proteins are a product of repeat-associated non-ATG translation from sense and antisense transcripts (Ash et al., 2013; Gendron et al., 2013; Mori et al., 2013a,b). Most frequent dipeptide repeat pathology, particular inclusions of poly (GP) or poly (GA) can be detected in neocortex, hippocampus and cerebellum but dipeptide repeat inclusions are also abundant in thalamus (Schludi et al., 2015).

These recent neuropathological and neuroimaging findings provide evidence for an underappreciated role of the cerebellum and thalamus in the pathogenesis of FTD and ALS caused by a repeat expansion in *C9orf72* (Prado et al., 2015; Rohrer et al., 2015; Schludi et al., 2015). We hypothesized that thalamus and cerebellum show an enhanced degree of atrophy in *C9orf72* expansion carriers compared to sporadic patients and that thalamic atrophy goes beyond the expected atrophy in the prefrontal and temporal subregion of the thalamus in *C9orf72* mutation carriers. In the current study, we therefore aimed to elucidate the regional brain atrophy focusing on thalamic and cerebellar atrophy in *C9orf72* mutation carriers compared to patients with sporadic FTD or FTD/ALS and healthy controls.

## Materials and Methods

### Ethics Statement

The study was performed according to the declaration of Helsinki (1991). Ethical approval for conduction of the study has been obtained at the coordinating site at the University of Ulm and all participating centers of the German consortium for frontotemporal lobar degeneration. Written informed consent was obtained from every participant.

### Subjects

A total of 77 participants from the cohort of the German consortium for frontotemporal lobar degeneration (Otto et al., 2011) were included in the study: 13 symptomatic *C9orf72* mutation carriers (8 FTD, 2 ALS, 3 FTD/ALS), 45 with sporadic FTD (Medford and Critchley, 2010) or FTD/ALS (Snowden et al., 2015) in whom a pathological *C9orf72* expansion, *MAPT* or *GRN* mutation has been excluded and 19 healthy elderly control subjects. Diagnosis was made according to current international consensus criteria (Brooks et al., 2000; Rascovsky et al., 2011). Demographic features of participants are listed in **Table 1**.

**Table 1:** Demographics and neuropsychological measures of the study sample.

	*C9orf72*	Sporadic	HC	*C9orf72* vs. HC	*C9orf72* vs. sporadic	sporadic vs. HC
N	13	45	19			
Gender (M/F)	8/5	26/19	12/7	n.s.	n.s.	n.s.
Positive family history (Y/N)	8/5	12/33	1/18	+	+	n.s.
Age	64.1 (8.5)	62.8 (9.4)	65.9 (10.1)	n.s.	n.s.	n.s.
Education	12.8 (2.7)	13.2 (3.3)	13.7 (3.1)	n.s.	n.s.	n.s.
Disease duration	2.5 (3.5)	3.2 (3.7)			n.s.	
MMSE	25.8 (3.1)	25.6 (4.1)	29.0 (0.9)	+	n.s.	+
FTLD-CDR-SOB	3.7 (1.0)	6.3 (2.9)	0.4 (0.6)	+	n.s.	+


*n.s., not significant; ^+^*p* < 0.05.*

Participants underwent general cognitive screening using the mini mental status examination (MMSE). To quantify the severity of dementia symptoms the FTD modified Clinical Dementia Rating Scale Sum of Boxes (FTD-CDR-SOB) (Knopman et al., 2008) score was used. Additionally age, education, disease duration and the occurrence of a positive family history were assessed.

### MRI Acquisition

All patients and controls underwent whole-brain T1-weighted MRI on 3T scanners, and on 1.5T scanners, where 3T scanning was not available. An array head coil with a minimum of 8 channels was used. 3D-MPRAGE sequences were acquired in sagittal orientation with 1 mm x 1 mm in-plane resolution, slice thickness 1 mm, and TR/TE = 2300/2.03 ms.

### MRI Data Processing and Volumetric Analysis

After pseudonymization and conversion from DICOM to ANALYZE 7.5 format the 3D T1-weighted images were processed by a fully automated and observer-independent method of atlas- and mask-based MRI volumetry using the Statistical Parametric Mapping 12 software (Wellcome Trust Centre for Neuroimaging, London, United Kingdom)^1^. The method has been described in detail elsewhere (Huppertz et al., 2010, 2016; Opfer et al., 2016) and was already applied to neurodegenerative diseases in various cross-sectional and longitudinal studies (Kassubek et al., 2011; Frings et al., 2012, 2014; Höglinger et al., 2014; Huppertz et al., 2016; Schönecker et al., 2016). In short, each T1 image was normalized to Montreal Neurological Institute (MNI) template space using diffeomorphic anatomical registration through exponentiated Lie algebra (DARTEL) (Ashburner, 2007) and segmented into gray matter, white matter, and cerebrospinal fluid components using the ‘unified segmentation’ algorithm of Statistical Parametric Mapping 12 with default parameters. The DARTEL algorithm is a highly elastic registration method resulting in a more precise registration of the individual brain to MNI space than the normalization methods in previous SPM versions, thereby also improving the adaptation to the space of the atlases used in the further post-processing. The volumes of specific brain structures and compartments were calculated by voxel-by-voxel multiplication and subsequent integration of normalized and modulated component images (gray matter, white matter or cerebrospinal fluid) with predefined masks in the same space. These masks are derived from different probabilistic brain atlases, such as the LONI Probabilistic Brain Atlas (LPBA40) provided by the Laboratory of Neuroimaging (LONI) at the University of California, Los Angeles, United States^2^ (Shattuck et al., 2008) and the probabilistic thalamic connectivity atlas provided by the Nuffield Department of Clinical Neurosciences at the University of Oxford, United Kingdom (Oxford Thalamic Connectivity Atlas; OTH)^3^ (Behrens et al., 2003). Target structures were chosen *a priori* for analysis of group differences in volume (13 in total, see **Table 2**). As our study aimed to determine the amount of thalamic and cerebellar atrophy of *C9orf72* mutation carriers compared to sporadic patients and HC, we included as regions of interest cerebellum, cerebellar vermis plane and pons as derived from structures and further parcellations in the LPBA40 atlas (Huppertz et al., 2016) and in addition all structures of the OTH atlas, i.e., overall thalamic volume as well as the primary motor, sensory, posterior parietal, occipital, temporal and prefrontal subregion of the thalamus that are connected to the corresponding cortical zone. Furthermore, since frontotemporal cortex shows pronounced atrophy in *C9orf72* mutation carriers as well as in sporadic patients the frontal and temporal cortex as derived from the integration of single gyri of the LPBA40 atlas (Huppertz et al., 2010) have been included as regions of interest as well.

**Table 2:** *Anatomical* structures selected for volumetric analysis per group mean and SD (in ml), and pairwise *post hoc* Bonferroni test results.

Dependent variable	*C9orf72*	Sporadic	HC	*C9orf72* vs. HC	*C9orf72* vs. sporadic	Sporadic vs. HC
Frontal	246.90 (26.96)	265.37 (33.10)	296.54 (15.82)	+	n.s.	+
Temporal	158.31 (11.97)	164.44 (18.78)	181.70 (14.08)	+	n.s.	+
Cerebellum	109.46 (9.82)	109.44 (11.43)	113.33 (10.24)	n.s.	n.s.	n.s.
Cerebellar vermis plane	949.72 (101.54)	973.68 (96.92)	1020.74 (86.26)	n.s.	n.s.	n.s.
Pons	15.30 (1.40)	15.23 (1.76)	15.25 (1.36)	n.s.	n.s.	n.s.
Thalamic regions						
Thalamus all	15.70 (1.49)	17.62 (1.62)	19.09 (1.50)	+	+	+
Primary motor	0.97 (0.08)	1.03 (0.07)	1.06 (0.08)	n.s.	n.s.	n.s.
Sensory	1.11 (0.09)	1.18 (0.09)	1.23 (0.09)	+	+	n.s.
Premotor	1.70 (0.15)	1.84 (0.14)	1.92 (0.14)	+	+	n.s.
Posterior parietal	3.09 (0.27)	3.35 (0.26)	3.50 (0.25)	+	+	n.s.
Occipital	1.41 (0.13)	1.62 (0.18)	1.77 (0.16)	+	+	+
Temporal	2.91 (0.29)	3.45 (0.48)	3.92 (0.43)	+	+	+
Prefrontal	4.52 (0.37)	5.14 (0.51)	5.70 (0.46)	+	+	+


*n.s., not significant; ^+^*p <* 0.05, corrected for multiple comparisons.*

### Statistical Analysis

Data were analyzed using SPSS23. Non-dichotomized mean scores of demographic and neuropsychological data were compared across the three groups (*C9orf72* mutation carriers, sporadic patients and HC) via Kruskal–Wallis test and Mann–Whitney test. Chi-square analysis was used to check for significant differences in gender and family history across all groups. Standard statistical significance level was set at *p* < 0.05.

For each region of interest the individual volume at clinic presentation was determined in ml. For comparison, the measured volumes were corrected by individual intracranial volume and standardized to the mean intracranial volume of healthy controls.

For group comparisons of volumetric data, a Kruskal–Wallis test was performed. Significance levels for the Kruskal–Wallis test were adjusted according to Bonferroni correction (*p* < 0.0038). Results of post-hoc tests were regarded significant if they survived an additional Bonferroni correction for multiple pairwise group comparisons. Spearman’s test was used to explore significant correlations between volumetric data and neuropsychological variables. Significance level was adjusted according to Bonferroni correction (*p* < 0.0038) as well.

Furthermore, to assess laterality of overall thalamic volume and thalamic subregions a laterality index (LI) defined as the ratio [(left - right)/(left + right)] was calculated (Seghier, 2008; Okada et al., 2016) for each study group. LIs can range from -1 to 1 with a positive LI indicating a leftward asymmetry. One-sample Wilcoxon signed rank tests were calculated to evaluate whether LIs were significantly different from zero. A Kruskal–Wallis test was performed for group comparisons of LIs. As for the volumetric analysis significance level was adjusted according to Bonferroni correction (*p <* 0.00625) and an additional *post hoc* Bonferroni correction was performed.

Parameters showing a significant difference between *C9orf72* mutation carriers and sporadic patients in the former analyses, i.e., overall thalamic volume and the volumes of the sensory, premotor, parietal, occipital, temporal and prefrontal subregion of the thalamus, the LI of the primary motor, sensory, premotor, occipital and prefrontal subregion of the thalamus as well as the neuropsychological parameters MMSE and FTD-CDR-SOB were subjected to a forward stepwise binary logistic regression to determine the best predictor of diagnosis. Furthermore, the receiver operating characteristic (ROC) curve was created to evaluate the utility of the model at distinguishing between *C9orf72* mutation carriers and sporadic patients.

## Results

### Demographics and Cognitive Scores

Demographics and cognitive scores of the study sample can be seen in **Table 1**. Participant groups did not differ in terms of gender, age, education and disease duration. Patient groups (*C9orf72* mutation carriers and sporadic patients) performed significantly worse at cognitive screening tests (MMSE and FTD-CDR-SOB) compared to HC but did not differ significantly from one another. *C9orf72* mutation carriers had, as expected, significantly more frequently a positive family history compared to sporadic patients and HC (see **Table 1**).

### Volumetric Analysis

**FIGURE 1:**
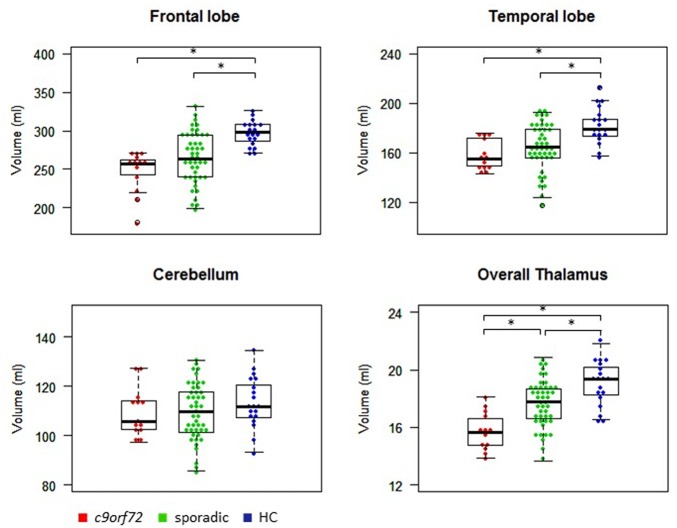
Volumes of frontal and temporal lobe, cerebellum and thalamus. ^∗^Indicates significant differences.

**FIGURE 2:**
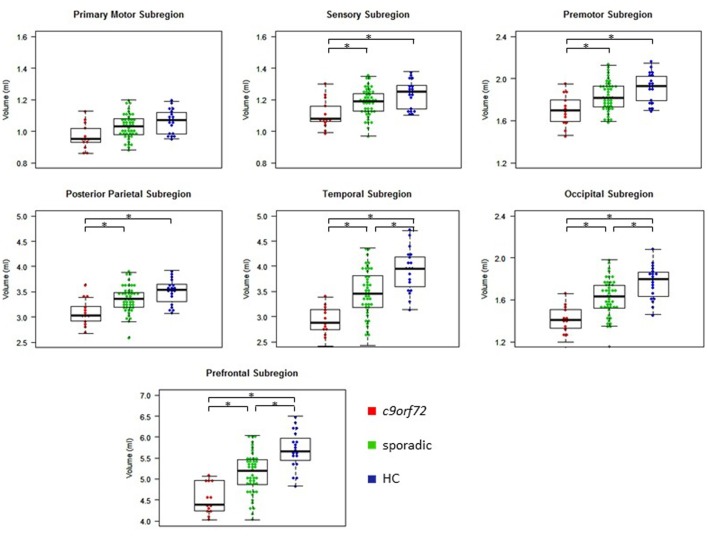
Volumes of thalamic subregions. ^∗^Indicates significant differences.

Kruskal–Wallis test revealed significant differences of the volumes of the frontal and temporal lobe, overall thalamic volume (**Figure 1**) and the volumes of the sensory, premotor, posterior parietal, occipital, temporal and prefrontal subregion of the thalamus (**Figure 2**). No significant differences could be detected for the cerebellum, the cerebellar vermis plane and pons as well as for the primary motor subregion of the thalamus (**Figures 1**, **2**).

*Post hoc* Bonferroni tests showed that sporadic patients compared to HC had reduced volumes of the frontal and temporal lobe as well as reduced volumes of overall thalamus and the occipital, temporal and prefrontal subregion of the thalamus (**Figures 1**, **2** and **Table 2**). Similarly, *C9orf72* mutation carriers had significantly smaller volumes of frontal and temporal lobe as well as all investigated thalamic subregions apart from the primary motor subregion (i.e., sensory, premotor, posterior parietal, occipital, temporal and prefrontal subregion of the thalamus) compared to HC. No significant differences of frontal and temporal lobe volumes could be detected between *C9orf72* mutation carriers and sporadic patients. However, although sporadic patients were somewhat more advanced, both in disease duration and in the FTD-CDR-SOB, *C9orf72* mutation carriers showed significantly smaller volumes of the sensory, premotor, posterior parietal, occipital, temporal and prefrontal subregion of the thalamus (**Figure 2** and **Table 2**).

### Correlation Analysis

**FIGURE 3:**
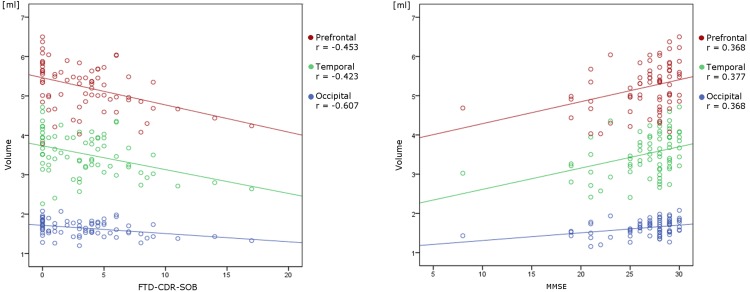
Linear correlation between volumetric data and neuropsychological variables. There is a significant negative/positive correlation of FTD-CDR-SOB/MMSE with the volumes of the prefrontal, temporal and occipital subregion of the thalamus.

Correlation analysis showed a significant positive/negative correlation of MMSE/FTD-CDR-SOB with overall thalamic volume (*r_s_* = 0.352, *r_s_* = -0.406), the volumes of the prefrontal (*r_s_* = 0.368, *r_s_* = -0.453), temporal (*r_s_* = 0.377, *r_s_* = -0.423) and occipital (*r_s_* = 0.367, *r_s_* = -0.385) subregion of the thalamus as well as with frontal (*r_s_* = 0.392, *r_s_* = -0.607) and temporal (*r_s_* = 0.364, *r_s_* = -0.391) lobe volume (**Figure 3**).

### Laterality Indices

Laterality indices were investigated in this study as well. All study groups showed positive LIs of the occipital, prefrontal, and posterior parietal subregion of the thalamus and significantly negative LIs of the primary motor, sensory, premotor and temporal subregion of the thalamus. Overall thalamic volume showed significantly positive LIs in sporadic patients and HC but was not significantly different from zero in *C9orf72* mutation carriers.

Kruskal–Wallis test revealed significant group differences of the LIs of overall thalamus and of the primary motor, sensory, premotor, occipital and prefrontal subregion of the thalamus. Only for the posterior parietal and the temporal subregion of the thalamus, no significant group differences could be detected. *C9orf72* mutation carriers differed significantly from sporadic patients in the aforementioned LIs. Furthermore LIs of overall thalamus, sensory, premotor, occipital and prefrontal subregion of the thalamus differed significantly between *C9orf72* mutation carriers and HC. No significant differences of LIs could be detected between sporadic patients and HC. For every investigated thalamic subregion as well as for overall thalamus, *C9orf72* mutation carriers showed the lowest LIs (**Table 3**).

**Table 3:** Laterality indices of overall thalamus and thalamic subregions, and pairwise *post hoc* Bonferroni test results.

Dependent variable	*C9orf72*	Sporadic	HC	*C9orf72* vs. HC	*C9orf72* vs. sporadic	Sporadic vs. HC
Overall thalamus	0.0061	0.0327	0.0349	+	+	n.s.
Primary motor	-0.0956	-0.0687	-0.0722	n.s.	+	n.s.
Sensory	-0.0731	-0.0494	-0.0503	+	+	n.s.
Premotor	-0.0795	-0.0531	-0.0548	+	+	n.s.
Posterior parietal	0.0360	0.0576	0.0525	n.s.	n.s.	n.s.
Occipital	0.2373	0.2698	0.2814	+	+	n.s.
Temporal	-0.0713	-0.0448	-0.0419	n.s.	n.s.	n.s.
Prefrontal	0.0366	0.0632	0.0690	+	+	n.s.


*n.s., not significant; ^+^*p* < 0.05, corrected for multiple comparisons.*

### Binary Logistic Regression

Target volumes, LIs and neuropsychological variables that showed a significant difference between *C9orf72* mutation carriers and sporadic patients were subjected to a forward stepwise binary logistic regression to determine the best predictor of diagnosis. The optimal logistic regression model using the volume of the prefrontal subregion of the thalamus (B = -2.54) and the LI of the occipital subregion of the thalamus (B = -24.81) as predictor variables (*p* < 0.05; Nagelkerke’s *R*^2^ = 0.441) resulted in an area under the curve (AUC) of 0.88 (95% CI: 0.80 – 0.97). The highest combination of sensitivity and specificity was acquired with a sensitivity of 1.00 and a specificity of 0.69 at a predicted probability cutoff of 0.14.

**FIGURE 4:**
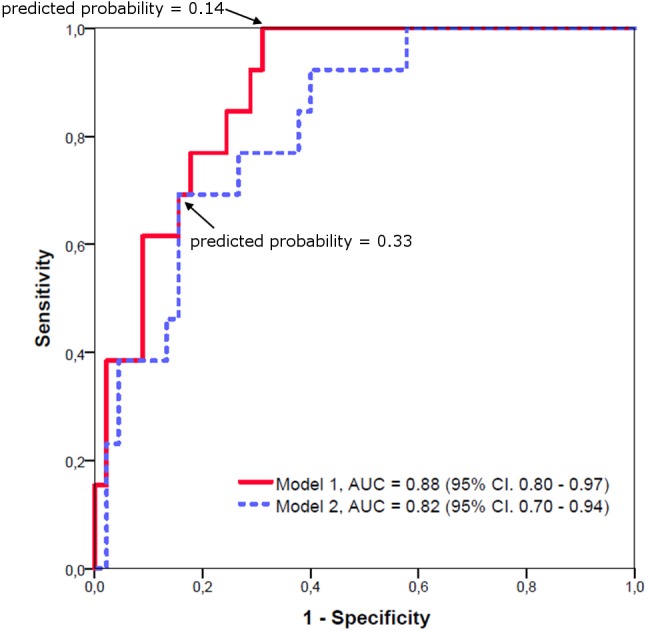
Receiver operating characteristic curves for logistic regression models of *C9orf72* mutation carriers versus sporadic patients. Model 1: Using the prefrontal subregion of the thalamus and the LI of the occipital subregion of the thalamus as predictor variables results in an AUC of 0.88. Model 2: Using only overall thalamic volume as predictor variable still results in an AUC of 0.82.

A logistic regression model using only overall thalamic volume as predictor variable (B = -0.82, *p* < 0.05; Nagelkerke’s *R*^2^ = 0.327) still resulted in an AUC of 0.82 (95% CI: 0.70 – 0.94). The highest combination of sensitivity and specificity was acquired with a sensitivity of 0.69 and a specificity of 0.84 at a predicted probability cutoff of 0.33. The ROC curves are shown in **Figure 4**.

## Discussion

Neuropathological data show that DPR protein aggregates are abundant in the thalamus (Schludi et al., 2015). This is in accordance with previous reports of significant thalamic atrophy in *C9orf72* mutation carriers (Sha et al., 2012; Mahoney et al., 2012; Irwin et al., 2013). Furthermore, an early affection of the thalamus in presymptomatic *C9orf72* mutation carriers has been shown (Rohrer et al., 2015). Here we show a significant volume reduction of certain thalamic subregions of symptomatic *C9orf72* mutation carriers compared to sporadic patients and HC and reveals overall thalamic volume to be a useful predictor of *C9orf72* mutation carrier status. The negative correlation of thalamic volume and clinical parameters highlights the important role of the thalamus in the pathogenesis of *C9orf72* associated clinical pictures of FTD and ALS.

*C9orf72* mutation carriers present more frequently with psychotic symptoms and show more severe memory impairment than sporadic patients (Boeve et al., 2012; Snowden et al., 2015). In our study cohort, disease severity measured by MMSE and FTD-CDR-SOB was not only correlated with the volumes of the frontal and temporal lobes but also with overall thalamic volume and the volumes of the prefrontal, temporal and occipital subregion of the thalamus, illustrating their clinical relevance. These subregions furthermore showed the most striking volumetric differences between *C9orf72* mutation carriers and sporadic patients. The occipital subregion includes lateral geniculate nucleus and parts of the inferior pulvinar, temporal subregion includes parts of the mediodorsal nucleus (MDN) and the medial and inferior pulvinar and the prefrontal subregion includes some of MDN, ventral anterior nucleus and anterodorsal and anteromedial nucleus (Behrens et al., 2003; Johansen-Berg et al., 2005).

The “Salience Network (SN)” (Seeley et al., 2007) is an intrinsic connectivity network that is activated in response to emotionally significant stimuli and contributes to complex brain functions like guidance of behavioral responses, production of subjective feelings and initiation of cognitive control (Menon and Uddin, 2010; Medford and Critchley, 2010). The SN is anchored in the dorsal anterior cingulate and anterior insula but also includes various subcortical structures. Comparable SN disruption despite contrasting atrophy patterns in *C9orf72* mutation carriers and sporadic patients has been described (Lee et al., 2014). Atrophy of the medial pulvinar nucleus that has prominent reciprocal connections to major hubs of the SN (Romanski et al., 1997) could only be detected in *C9orf72* mutation carriers. As medial pulvinar nucleus atrophy predicted reduced SN connectivity, a strategic atrophy of the medial pulvinar nucleus has been proposed to contribute to SN disruption in *C9orf72* mutation carriers (Lee et al., 2014). We therefore hypothesize that especially atrophy of the medial pulvinar nucleus may have led to the detected volume reduction of the temporal subregion of the thalamus of *C9orf72* mutation carriers in our study cohort.

Another thalamic node that is part of the SN is the MDN. Studies detected significant atrophy of MDN in early stages of FTD (Seeley et al., 2008). MDN is believed to be involved in the pathogenesis of schizophrenia (Young et al., 2000; Alelú-Paz and Giménez-Amaya, 2008) and has been shown to play an important role in working memory and episodic memory (Gaffan and Parker, 2000; Watanabe and Funahashi, 2012; Mitchell and Chakraborty, 2013). It is involved in memory formation and influences emotional connotations also via extralemniscal pathways, e.g., connecting to the amygdala. For ALS a more frequent bulbar onset in mutation carriers is discussed. With view to FTD, mutation carriers may present with more psychosis displaying delusions and hallucinations and/or catatonic features. Also, late-onset dementia and depressive syndromes with cognitive impairments were reported (Ducharme et al., 2017). We hypothesize that MDN atrophy is reflected by the volume reduction of the temporal and prefrontal subregion of the thalamus of *C9orf72* mutation carriers and that MDN atrophy leads to a disruption of SN and thereby contributes to the distinct clinical characteristics of mutation and non-mutations carriers.

Several studies have reported greater occipital (Boxer et al., 2011; Khan et al., 2012; Whitwell et al., 2012) and parietal (Sha et al., 2012; Whitwell et al., 2012) volume loss of *C9orf72* mutation carriers compared to sporadic patients. The detected volume loss of the occipital, posterior parietal and sensory subregion of the thalamus of *C9orf72* mutation carriers may therefore be due to a common degeneration of functionally connected regions. The fact that the atrophy pattern in our *C9orf72* mutation carrier study group goes beyond the expected atrophy in the frontal and temporal subregion of the thalamus is an indicator that atrophy in mutation carriers may exceed the SN and is in keeping with the described cortical pattern of atrophy detected in mutation carriers that also goes beyond the sporadic FTD-associated atrophy pattern.

In a recent volumetric MRI study, a classification accuracy of 93% could be obtained to discriminate between *C9orf72* mutation carriers, *MAPT* and *GRN* mutation carriers and sporadic patients by using 26 regional volume and asymmetry scores (Whitwell et al., 2012). A more conservative model requiring 14 variables was able to classify 74% of patients correctly. In contrast to the aforementioned study, we compared only *C9orf72* mutation carriers and sporadic patients. As group sizes differed in our study cohort, classification accuracy cannot directly be compared. Furthermore, as multicollinearity was present in our data, results of binary logistic regression have to be interpreted with caution. However, multicollinearity does not bias the result of logistic regression, but only affects calculations regarding individual predictor variables (Midi et al., 2010). The optimal logistic regression model resulted in an AUC of 0.88 while a logistic regression model using only overall thalamic volume as predictor variable still resulted in an AUC of 0.82. Both AUCs correspond to very good diagnostic accuracy (Šimundić, 2008). Our data therefore provide evidence of a combination of the volume of the prefrontal subregion and the LI of the occipital subregion of the thalamus and overall thalamic volume respectively to be of high predictive value in identifying *C9orf72* mutation carriers. MRI volumetry, especially of subcortical regions of interest, may therefore help to differentiate between *C9orf72* mutation carriers and sporadic patients, regardless of the presence of a positive family history. This is particularly useful since prediction of mutation status is extremely difficult based on clinical features alone. Nonetheless, the addition of clinical information like prominent psychosis or memory impairment (Boeve et al., 2012; Snowden et al., 2015), co-occurring FTD and ALS symptoms (DeJesus-Hernandez et al., 2011) and a positive family history may further improve prediction.

As in previous studies examining thalamic asymmetries in control subjects, we were able to detect left greater than right asymmetry in our HC (Flaum et al., 1995; Okada et al., 2016). Leftward asymmetry could also be detected in sporadic patients. Mainly the posterior parietal, occipital and prefrontal subregion of the thalamus seem to contribute to the detected overall leftward asymmetry, whereas the primary motor, premotor, sensory and temporal subregion of the thalamus display a rightward asymmetry. In contrast, although each thalamic subregion showed either rightward or leftward asymmetry, no significant overall thalamic asymmetry could be detected in *C9orf72* mutation carriers. This is consistent with the symmetric cortical atrophy pattern detected in *C9orf72* mutation carriers (Mahoney et al., 2012; Whitwell et al., 2012).

Although abundant DPR pathology within granule cell layer of the cerebellum seems to be a consistent finding in *C9orf72* mutation carriers (Al-Sarraj et al., 2011; Irwin et al., 2013) and a number of studies have reported more prominent cerebellar atrophy in *C9orf72* mutation carriers compared to sporadic patients (Mahoney et al., 2012; Whitwell et al., 2012; Irwin et al., 2013), we were not able to detect significant group differences with respect to cerebellar volume. In a recent study, focal atrophy localized to lobule VIIa-Crus I in the superior-posterior region of the cerebellum could be detected in *C9orf72* mutation carriers compared to HC (Bocchetta et al., 2016). As this area is connected via the thalamus to the prefrontal cortex (Krienen and Buckner, 2009; Stoodley and Schmahmann, 2010) and therefore with the SN (Caulfield et al., 2016) and is associated with goal-directed behaviors, its involvement in *C9orf72*-associated FTD and ALS seems plausible. Perhaps an investigation of cerebellar subregions would have revealed atrophy clusters specific for *C9orf72* mutation carriers.

A limitation of the current study that needs to be considered is the small number of *C9orf72* mutation carriers enrolled (N = 13) which rendered subdividing *C9orf72* mutation carriers in FTD, FTD/ALS and ALS patients impossible. Further studies of larger cohorts subdividing the different *C9orf72* mutation carrier phenotypes are necessary. Furthermore multi-scanner data-sets and scans performed on 3T scanners and on 1.5T scanners have been pooled in the analyses. However multi-site studies offer a good possibility to investigate rare disorders like neurodegenerative diseases caused by *c9orf72* mutation carrier status. Another limitation of our study was the absence of a neuropathological confirmation of our sporadic patient study group which leaves the possibility that a percentage of cases had a mismatch of clinical diagnosis and underlying pathology.

Keeping these limitations in mind, our findings reveal a combination of the volume of the prefrontal subregion and the LI of the occipital subregion of the thalamus and overall thalamic volume respectively to be useful predictors of mutation carrier status. We furthermore demonstrated that the thalamic atrophy pattern in *C9orf72* mutation carriers goes beyond hubs of the SN and is in good agreement with the cortical atrophy pattern detected in *C9orf72* mutation carriers, indicating a retrograde degeneration of functionally connected regions.

## Author Contributions

SS and JL coordinated and drafted the manuscript. CN, JD-S, MO, AL, JaK, BL, SA-S, ES, KF, AS, JoK, MM, JP, HJ, TB-B, MS, and CP were involved in the imaging acquisition. AV performed the genetic testing. SS, CV, and H-JH were involved in the data analysis. SS, JF, TA, DE, BF, MD, JoK, MS, AD, TK, and JL were involved in the interpretation of data. All authors critically revised the manuscript and read and approved the final manuscript.

## Conflict of Interest Statement

The authors declare that the research was conducted in the absence of any commercial or financial relationships that could be construed as a potential conflict of interest. The handling Editor declared a past co-authorship with one of the authors JL.

## Footnotes

1 www.fil.ion.ucl.ac.uk/spm

2 http://www.loni.usc.edu/atlases/

3 http://fsl.fmrib.ox.ac.uk/fsl/fslwiki/Atlases

## Abbreviations

ALS: amyotrophic lateral sclerosis
AUC: area under the curve
DPR: dipeptide repeat proteins
FTD: frontotemporal dementia
FTD-CDR-SOB: FTD modified Clinical Dementia Rating Scale Sum of Boxes
HC: healthy controls
LI: laterality index
LPBA40: LONI Probabilistic Atlas
MDN: mediodorsal nucleus
MMSE: mini mental status examination
OTH: Oxford Thalamic Connectivity Atlas
ROC: receiver operating characteristic
SN: salience network
